# Exploiting the advantages of a wireless seed localization system that differentiates between the seeds: Breast cancer resection following neoadjuvant chemotherapy

**DOI:** 10.1002/cnr2.1690

**Published:** 2022-08-08

**Authors:** Elizabeth Shaughnessy, Charmi Vijapura, Chantal Reyna, Jaime Lewis, Kyle Lewis, Su‐Ju Lee, Lawrence Sobel, Rifat Wahab, Lauren Rosen, Ann Brown

**Affiliations:** ^1^ Department of Surgery, Section of Breast Surgery within Division of Surgical Oncology University of Cincinnati Cincinnati Ohio USA; ^2^ Department of Radiology, Division of Breast Imaging University of Cincinnati Cincinnati Ohio USA; ^3^ Department of Surgery Crozer Health System Springfield Pennsylvania USA; ^4^ Department of Pathology, Section of Surgical Pathology within Division of Anatomic Pathology University of Cincinnati Cincinnati Ohio USA

**Keywords:** breast localization, tagged axillary dissection, tagged lymph node, wireless localization

## Abstract

**Background:**

Most wireless localization methods utilize only one means of detection for the surgeon, sufficient to localize a single small breast lesion for excision. Complex cases requiring bracketing of a larger lesion or localization of two or more close lesions can superimpose the signal from separate “seeds” with such methods. The lack of discernment between the localization “seeds” can disorient the surgeon, risking a missed lesion on excision and longer operative times. with the use of neoadjuvant chemotherapy prior to breast surgery, the necessity of localizing both a breast lesion and an axillary lymph node previously biopsied is becoming frequent.

**Case:**

A 44 year‐old woman underwent neoadjuvant chmotherapy for a breast cancer the did not express estrogen receptor, progesterone receptor, or HER2 receptor. In establishing the extent of disease, a suspicious ipsilateral lymph node was biopsied and found to contain metastatic disease. She had an excellent response to the chemotherapy, with decreased size of the primary tumor and the previously biopsied lymph node. The patient desired breast conservation. The primary tumor and associated calcifications were bracketed using two different Smartclips™, with a third localizing the lymph node biopsied.

**Conclusion:**

This report illustrates how the use of three SmartClips™, within the EnVisioTM system, allowed for separate tracking of each “seed” throughout a complex surgery in a patient following neoadjuvant chemotherapy. This resulted in successful resection of both the tumor and the tagged lymph node.

## 1 INTRODUCTION

The widespread use of screening mammography has led to the diagnosis of increasingly smaller and non‐palpable lesions in the breast. This presents a dilemma to the consulting surgeon. Prior to screening, excisional breast biopsies were based on palpation. Without haptic guidance, the placement of a needle and wire with imaging guidance on the morning of surgery became the method of choice for preoperative localization as it was widely available and relatively inexpensive. In the case of more extensive disease, multiple wires were frequently placed in the breast to localize larger or multiple areas, and wires were rarely placed in the axilla for localization of lymph nodes.

Wire localization has its limitations and challenges, including migration or displacement of the wire prior to a patient's arrival in the operating suite^1^ and fragmentation during surgery.^2^ Pain experienced by patients during wire leads to vasovagal reactions in a reported 7% of wire localizations^3^ and discomfort caused by the wire may contribute to wire migration and displacement prior to surgery as patients fidget and reposition. Localization wires are placed the same day of surgery to minimize the risk of migration, which limits the flexibility of scheduling for radiologic procedures and operative procedures. Difficulties in localizing the lesion can delay operative start times^4^ and failure to identify the lesion in the intraoperative specimen radiograph can prolong surgery.^5^

Intraoperatively, the success of wire localization depends on the surgeon's mental reconstruction of the images, the trajectory of the wire, and the perceived intraoperative position of the lesion.^6^, ^7^ Approximately 2.5% of wire localizations are unsuccessful. Factors impacting poor surgical outcome include multiple lesions, small size of the targeted lesion, association with extensive calcifications, and small size of the surgical specimen.^8^

Wireless “seed” localization techniques have been developed to allow for greater flexibility in beginning an early operative day incorporating localization. This uncouples the tight time reliance of the surgeon on the radiologist's performance of the wire localization prior to the surgical resection. These newer “seed” methods include but are not limited to the use of a radioactive iodine‐125 seed, a radiofrequency identification tag, the SAVI® Scout (Cianna Medical, Inc.; Aliso Viejo, CA) implantable non‐radioactive infrared‐activated electromagnetic wave reflector, or one that utilizes magnetic detection (Magseed®; Endomagnetics, Inc.; Austin, TX). Multiple series have described the utility of these approaches in excising non‐palpable breast lesions.^8^, ^9^, ^10^, ^11^, ^12^ However, these series do not address the successful use of multiple seeds in a patient's resection.

With the increased utilization of neoadjuvant chemotherapy for breast cancers, newer algorithms for application of axillary sentinel lymph node biopsy are evolving in the management of the patient who presents with cN1 disease that converts to cN0 following chemotherapy.^13^, ^14^ Consequently, surgical institutions are now frequently localizing a biopsied positive lymph node in the axilla. Separate from this challenge, larger lesions of DCIS or multifocal lesions that require placement of more than one seed for localization prior to excision create yet another hurdle in the use of “seed” techniques. Combining the breast localization with axillary node localization adds another level of complexity to the surgery following neoadjuvant chemotherapy with cN1/2 evolving to cN0. The “seeds” must be sufficiently differentiated from each other to maximize the potential for negative margins and delivery of biopsied positive lymph nodes for final pathologic staging. The recent development of the SmartClip™ for the EnVisio System™ (Elucent Medical, Inc.; Eden Prairie, Minnesota) has provided a non‐radioactive and wireless mechanism by which up to three sites can be localized. This case report describes a test of this system for discernment of individual SmartClips™ using the maximal number, with one placed in a biopsied positive axillary lymph node and two placed in the breast to bracket a large nonpalpable lesion following neoadjuvant chemotherapy.

## 2 CASE PRESENTATION

The University of Cincinnati database on breast disease, approved by the Institutional Review Board, includes data relative to breast cancer and its treatment (IRB 2019 ‐0147). Separate consent was obtained from this single patient for case report purposes. UC Health switched to use of the SmartClip™ in localizations of the breast and axilla in 2019. The application of this localization technique can include biopsy‐proven breast cancers, benign lesions, as well as lymph nodes.

This patient is a 44‐year‐old woman, originally from the Ivory Coast, who had was found to have a benign cyst in the left breast on her first screening mammogram the year previous. Her screening mammogram in July 2020 revealed new calcifications. Anterior to these calcifications was the previously described cyst, unchanged. Diagnostic mammogram of the left breast at that time showed a 1.4 cm grouping of pleomorphic calcifications in the upper outer quadrant. Ultrasound of the left breast was performed for further evaluation due to the patient's breast density, which also demonstrated an irregular 3.6 cm mass associated with the calcifications. Ultrasound of the left axilla demonstrated a suspicious left axillary lymph node. The suspicious left breast mass and lymph node underwent ultrasound‐guided core needle biopsy. Screening and diagnostic workup was performed at the Breast Imaging Center within the Barrett cancer Center at the University of Cincinnati Medical Center, in Cincinnati, Ohio, USA. Pathology revealed primary invasive ductal carcinoma associated with high‐grade ductal carcinoma in situ in the breast, as well as carcinoma metastatic to the biopsied lymph node. Tissue markers were left in both the diagnosed cancer as well as the affected lymph node at the time of biopsy.

The pathology of the left breast primary cancer revealed invasive ductal carcinoma, high grade. The receptor profile of this patient's tumor proved negative for estrogen receptor, progesterone receptor and HER2/neu receptor expression, with 50% proliferation inferred by Ki‐67 immunohistochemical staining. She was referred for consultation with a surgical oncologist as well as a medical oncologist. She stated that she had no symptoms relative to feeling a new mass, any focal pain, or nipple discharge. She had no reported weight loss and enjoyed a high quality of life. She had no symptoms to suggest distant metastatic disease, and her CT scans of chest, abdomen and pelvis failed to demonstrate any metastasis. On physical exam, the mass measured at least 4.0 cm, including overlying skin, with use of external calipers. An enlarged, mobile left axillary node was appreciated also. Given the size of the tumor and the triple negative receptor profile, the standard of care strongly supported initiation of neoadjuvant chemotherapy prior to surgery.

The patient underwent a medical port insertion for ease in venous access for treatment. She received four doses of doxorubicin and cyclophosphamide, cycling every 2 weeks, followed by eight weekly doses of paclitaxel. She returned for evaluation prior to surgical resection as she completed her regimen. She demonstrated a remarkable clinical improvement, with no palpable mass or palpable ipsilateral lymph node at the completion of chemotherapy. Repeat imaging confirmed marked decrease in size of the breast mass and the known affected lymph node, with no appreciable change in the extent of the suspicious calcifications. Since she desired breast conservation, we proceeded to prepare for a left partial mastectomy in addition to left axillary sentinel lymph node biopsy. In addition, a targeted axillary dissection of the previously biopsied lymph node (tagged) would be included, as it had proven to be positive for metastatic breast cancer cells prior to neoadjuvant therapy. A completion axillary lymph node dissection would follow if she should have any tumor cells within the lymph nodes excised.^13^

Inclusion of the previously tagged positive lymph node in the resection, if proven to be a sentinel lymph node as well, would lower the false negative rate in the context of the sentinel lymph node biopsy.^14^ Consequently, we chose to localize the tagged node with a SmartClip™ as a targeted axillary dissection, as well as bracket the residual malignant calcifications within the left breast with two additional SmartClips™.

### 2.1 SmartClip™

**FIGURE 1:**
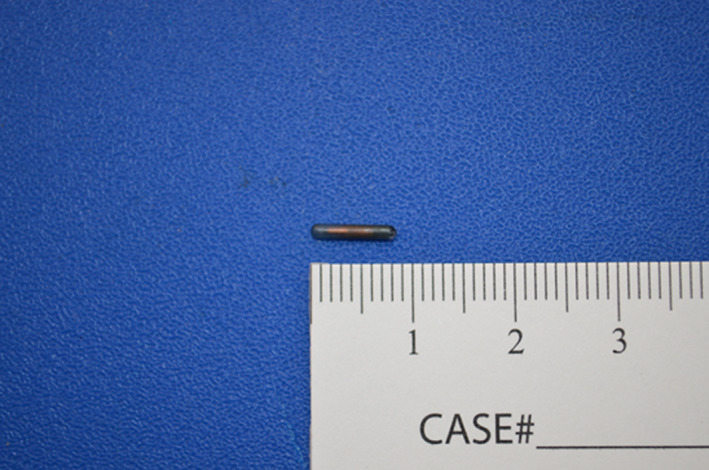
Relative size and shape of a SmartClip™

The SmartClip™ is a fiducial composed of a non‐radioactive ferrite core with parylene C coating, measuring 8 mm in length and 1.4 mm in diameter (Figure 1). Each SmartClip™ is preloaded into a 15‐gauge 10‐cm long SmartClip™ Lite applicator device; one each of the three different colors and shapes that appear on the display–green circle, pink square, or purple triangle. The applicators have a sharp, beveled end for skin entry; the SmartClips™ were designed to be placed inside tissue. Once placed, SmartClips™ can be differentiated intraoperatively with the guidance of the display, with the operating surgeon choosing a field to pursue any one of the colored shapes. For this case, the radiologist had chosen the applicators with three different identifying colors and shapes on the EnVisio™ Navigation heads up display (HUD) system, evident once the patient pad is activated with the patient on the operating table. SmartClip™ is approved by the U.S. Food and Drug Administration for potential permanent implantation in soft tissue; it could be placed as the tissue marker at the time of biopsy. A maximum of three different SmartClips™ can be implanted per patient at this time. If this patient had a pacemaker, then the use of the SmartClip™ would be contraindicated.

### 2.2 Localization technique

Several days before the planned surgical resection, the patient presented to the Breast Imaging Center, where insertion of two different SmartClips™ were placed to bracket the malignant calcifications, and a third Smartclip™ inserted within the previously biopsied lymph node identified by a tissue marker. These localizations were performed by a breast radiology fellow under the direct supervision of a dedicated breast radiologist. Aseptic technique was used.

**FIGURE 2:**
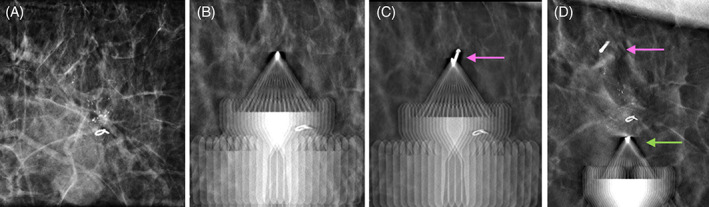
Images of single‐view bracketed SmartClip™ localization of the malignant mass and calcifications using a superior approach in the craniocaudal view. (A). Tomosynthesis facilitates targeting and insertion of the applicator to the posterior margin of the calcifications, (B), and deployment of the Pink SmartClip™ (pink arrow), (C), immediately followed by placement of the Green SmartClip™ applicator (green arrow) along the anterior margin, D

First, the bracketed localization of the malignant mass and calcifications in the left breast was performed using tomosynthesis guidance utilizing a single‐view superior approach (Figure 2). The patient was positioned in the upright Hologic Affirm® mammography unit (Hologic, Inc., Marlborough, MA). Local anesthetic was injected at each planned localization site prior to insertion. Initially, the posterior and superior margin was targeted on scout tomosynthesis images. A Pink SmartClip™ applicator was inserted through two sterile plastic guides into the breast tissue to the appropriate depth. Repeat tomosynthesis breast images confirmed the bevel of the SmartClip™ applicator in the targeted position (Figure 2B) and the SmartClip™ was deployed (Figure 2C). Next, the anterior‐inferior margin was targeted on tomosynthesis images. A Green SmartClip™ applicator was inserted through the guides into the breast tissue at this anterior site to the targeted depth. Repeat tomosynthesis images confirmed the position of the applicator tip (Figure 2D) and the Green SmartClip™ was deployed.

**FIGURE 3:**
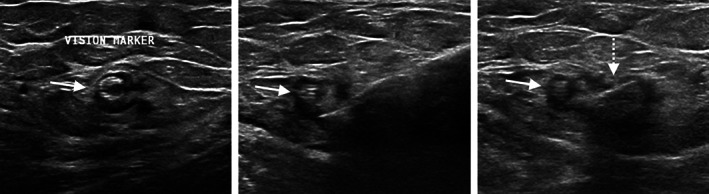
Ultrasound images during SmartClip™ localization of the tagged left axillary lymph node containing a Tumark Vision™ marker (solid arrow). (A). The applicator is placed into the lymph node with the bevel adjacent to the marker, (B), and the Purple SmartClip™ is deployed (dashed arrow), C

Lastly, ultrasound localization was performed for the tagged metastatic left axillary lymph node containing a Tumark® Vision tissue marker placed at the time of prior biopsy (Figure 3A) (Hologic, Inc.; Marlborough, MA). The patient was positioned supine in the ultrasound biopsy suite with her left arm overhead. Following local anesthesia, a Purple SmartClip™ applicator was inserted toward the target under direct sonographic guidance. The SmartClip™ was deployed once the applicator tip was visualized within the node (Figure 3B, C).

### 2.3 Operative technique

Several days after localization, the patient returned for surgical management in late December 2020. Two hours prior to surgery, the patient underwent peri‐areolar injection of 0.425 mCi of 99 m‐technetium sulfur colloid of the left breast in the Same Day Surgery area. This was massaged by the patient to enhance its migration to the ipsilateral axilla. After the patient was brought to the operating suite, she was administered an inhaled general anesthetic. The arm ipsilateral to the breast cancer was placed on an armboard at slightly less than 90° to her torso. Sterile methylene blue dye (5 ml of 1.0% concentration) was placed in a syringe with 15 ml sterile injectable saline solution. After she was cleansed and draped, the syringe of methylene blue dye with saline was injected subcutaneously under her left areola at four equidistant points. This was then massaged for 10 min.

The EnVisio™ Navigator was placed on the electrocautery pen; through interaction with the “pad” under the patient, its use locates the position of the SmartClip™‐tagged axillary lymph node, or either of the two SmartClips™ bracketing the cancer, depending on which SmartClip™ is chosen for primary detection. The “Heads Up Display” (HUD) screen was mounted pre‐operatively on the drape pole to the patient's right, across from the operating surgeons. Detection of the SmartClips™ was confirmed on the EnVision View (**Fig**ure 4) of the HUD. Prior to planning incisions, the locations of the SmartClips™ within the breast were marked on the patient's using a skin marker, both anteriorly and laterally. An incision was planned for the breast cancer separate from the incision within the axilla.

**FIGURE 4:**
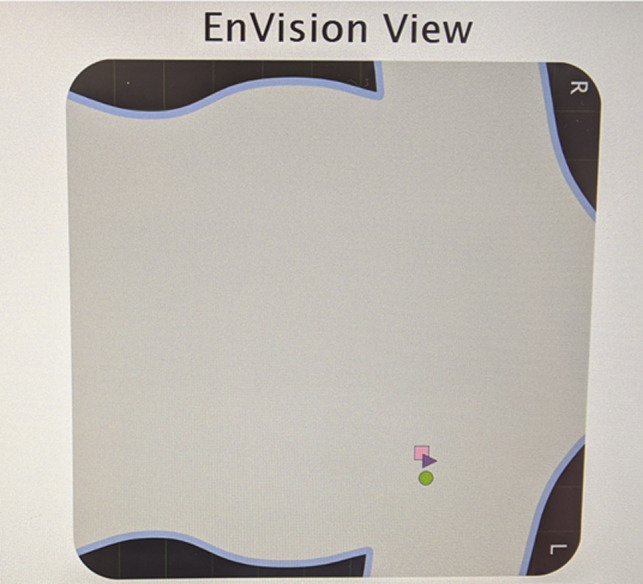
Bracketed residual left breast cancer with Pink (square) SmartClip™ posterior to the lesion, Green (circle) SmartClip™ anterior to the lesion, and Purple (triangle) SmartClip™ targeting the node. The patient's larger breast size overlapped the axilla at rest

**FIGURE 5:**
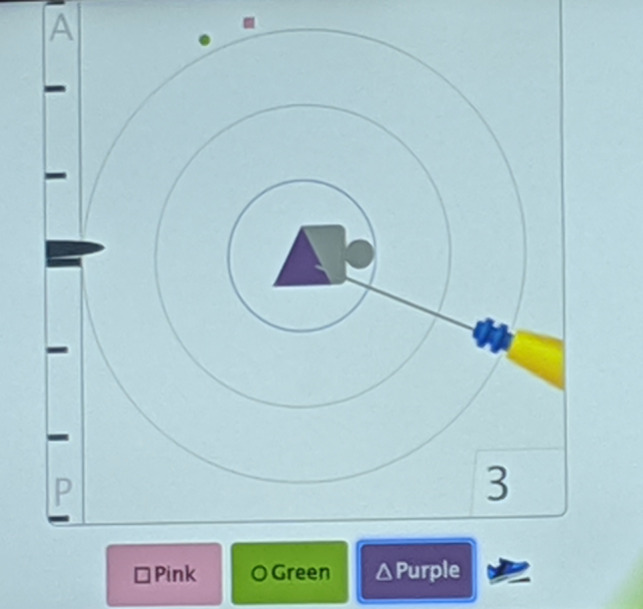
The orientation of the patient is depicted by the small gray circle “head” relative to the gray boxed “torso” central within the concentric rings. Using the foot pedal, the Purple (triangle) SmartClip™ was chosen for localized resection. The left margin indicates the relative depth (anterior to posterior) of the electrocautery tip to the targeted SmartClip™. The target circles indicate planar distance from the target in centimeters. The numeral in the lower right indicates the direct distance in millimeters to the chosen SmartClip™ target—here 3 mm. The pictured electrocautery tip is pointing to the Purple SmartClip™ within the tagged node

The sentinel lymph node biopsy and targeted axillary dissection were performed first. An incision was planned under the left axillary hairline for a length of approximately 3 cm. After the incision was made and extended through the dermis, the location of the SmartClip™ tagged node was easily checked for depth and relative location on a regular basis since the electrocautery and Navigator are attached (Figure 5). The HUD screen provides two‐dimensional distances from the axial position of the SmartClip™ as well as the additional depth once within 3 cm along a Y‐axis to the left of the two‐dimensional distances. A sound indicator is an option many use as well; the frequency increases with proximity to the SmartClip™. The absolute distance of the Navigator to the SmartClip™ is provided in millimeters in the lower right corner of the HUD. In this patient's case, the sentinel lymph nodes were relatively superficial and just under the axillary fascia. The tagged node with the Purple SmartClip™ also contained the radiotracer and methylene blue dye, indicating that is also a sentinel lymph node. This node was carefully excised, avoiding large blood vessels or nerves. An intraoperative specimen radiograph confirmed removal of the tagged lymph node. This was forwarded to Surgical Pathology for intraoperative frozen section, while pursuing the additional sentinel lymph nodes. We received word of the first node containing treatment effect and residual metastatic cancer cells. A completion axillary node dissection was subsequently performed as part of standard of care for residual disease in the axilla.^13^

**FIGURE 6:**
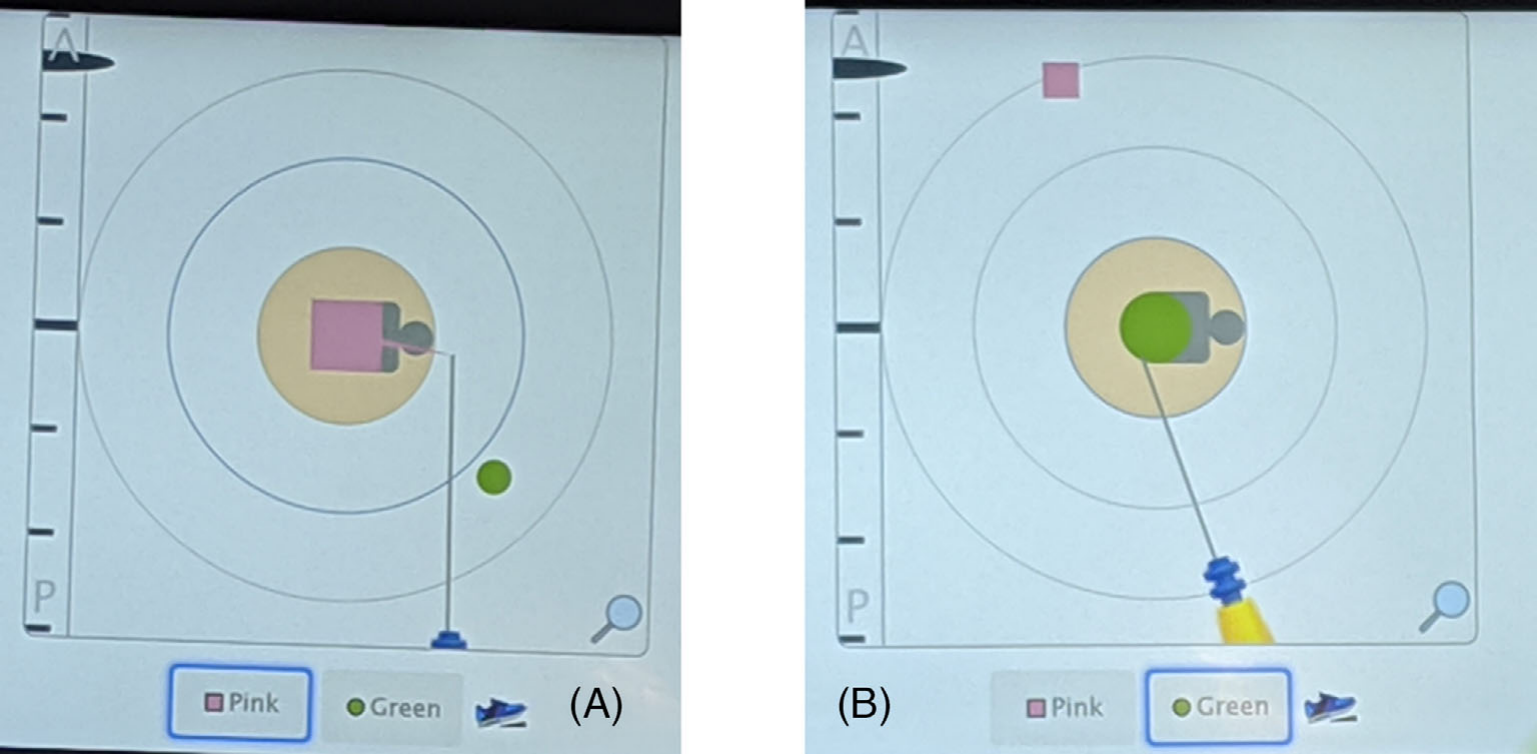
Resection of a SmartClip™‐bracketed lesion allows the surgeon to toggle between the two SmartClip™ images on the HUD; the SmartClips™ are actually physically placed along the posterior and anterior aspects of the lesion in this case. Relative distance to the other SmartClip™ can be visualized as denoted by the small geometric figure that is not concentrically located. A denotes the posterior Pink SmartClip™ and B denotes the anterior Green SmartClip™

A radial incision was planned for the left partial mastectomy in the lateral left breast at approximately the 2:30 position. Initially, the HUD demonstrated that the Pink and Green SmartClips™ were close from an anterior perspective as they appeared superimposed in position over the detection cassette under the pad beneath the patient's back (Figure 4). However, they were separated in depth, marking the anterior and posterior extent. With completion of the sentinel lymph node biopsy, the breast was shifted medially with assistance to separate the two SmartClips™ images from the anterior perspective to better visualize the SmartClips™ distinctly on the HUD. After making the incision for the lumpectomy, the surgeon toggled the foot pedal to alternate the focus on the HUD screen between the Pink (square) SmartClip™ and the Green (circle) SmartClip™ (Figure 6 A, B), specifically to reflect which SmartClip™ was closest in the dissection of this bracketed specimen. The distance from either clip to the outer aspect of the specimen was planned pre‐operatively, and the HUD easily guided the surgeon in attaining this margin. In this manner, both SmartClips™ were removed in one specimen, with the intervening tissue *en bloc*. An intraoperative specimen radiograph was obtained separately for the breast specimen and the images were forwarded to the breast radiologist for confirmation.

## 3 RESULTS

**FIGURE 7:**
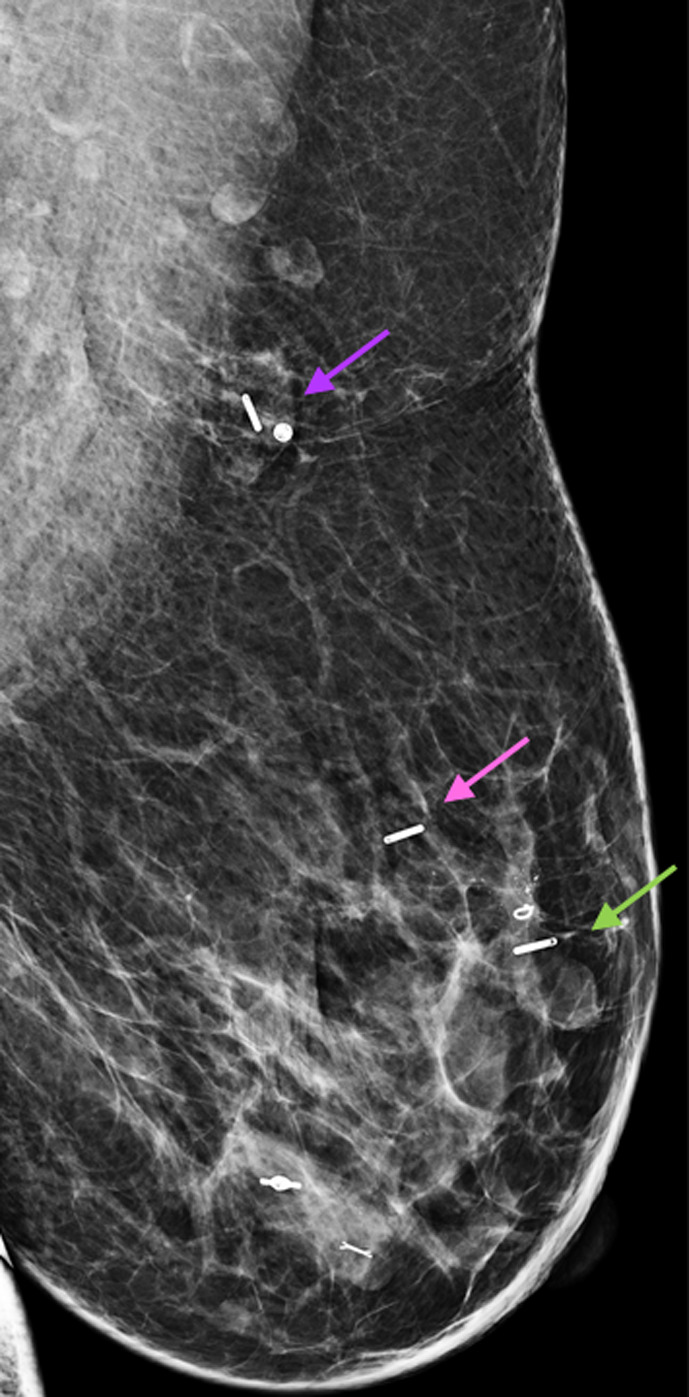
Post‐procedure ML projection of the left breast demonstrates SmartClip™ localization of the tagged node (purple arrow) and SmartClip™ bracketed localization of the posterior (pink arrow) and anterior (green arrow) extent of the malignant mass (Tumark Q™ marker) and calcifications. Two additional benign biopsy markers are present in the lower breast, which were not targeted for excision

The pre‐operative localization of the previously biopsied left axillary lymph node with a SmartClip™, as well as the use of two Smartclips™ to bracket the extent of left breast cancer using techniques similar to other seed methods, proceeded without difficulty. A final post‐procedure mammogram confirmed adequate position of all three SmartClips™ relative to their targets (Figure 7).

**FIGURE 8:**
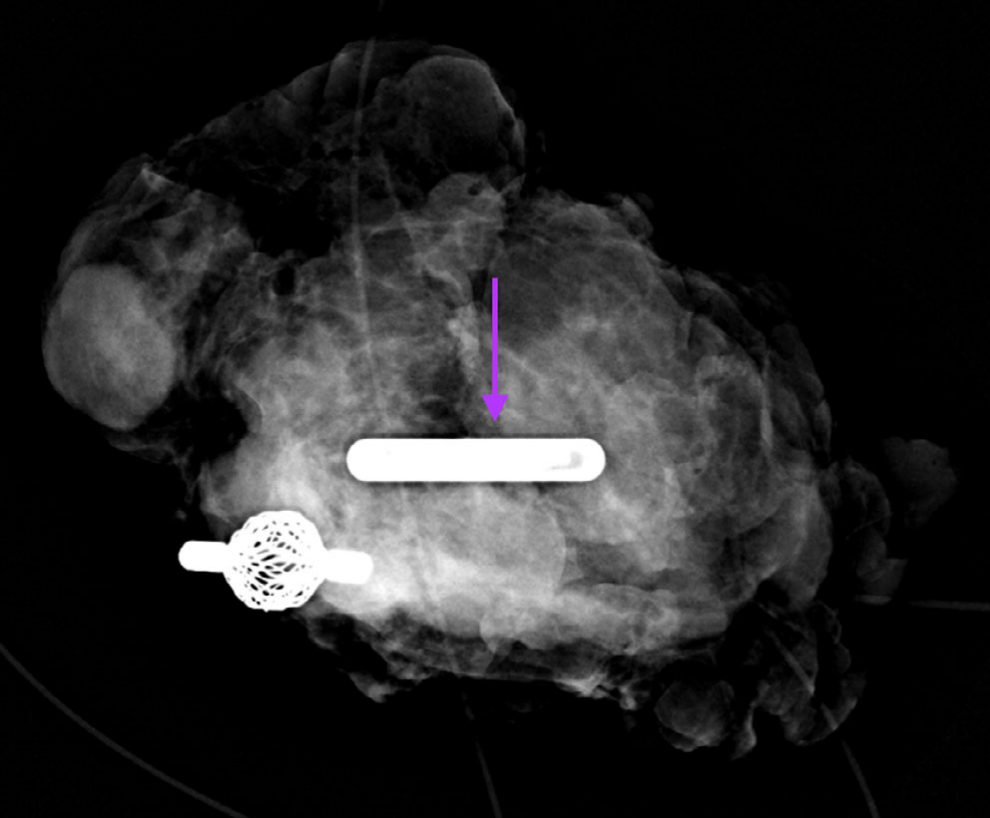
Specimen radiograph of the tagged axillary dissection node, revealing the tagged metastatic lymph node with biopsy marker and the purple SmartClip™ (arrow) used for localization

The first left axillary sentinel lymph proved to be the lymph node that initially contained metastatic disease prior to neoadjuvant chemotherapy. This was confirmed by intraoperative specimen radiograph of the lymph node, which also revealed the tissue marker and the SmartClip™ (Figure 8 **). I**ntraoperative frozen section of this first node demonstrated residual metastatic disease, which was sufficient to prompt the completion left axillary dissection.^13^

**FIGURE 9:**
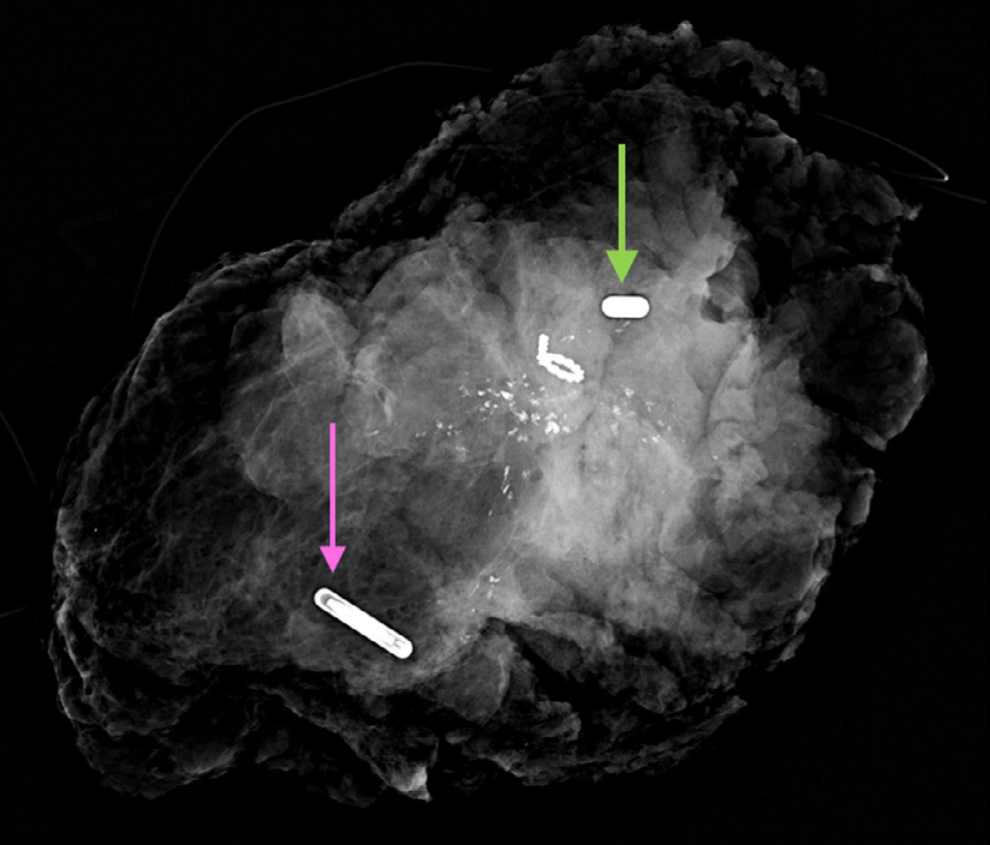
Radiograph of bracketed lumpectomy specimen demonstrates the malignant calcifications with biopsy marker and the Pink and Green SmartClips™ (arrows) used for the posterior and anterior localization respectively

The bracketed SmartClip™‐localized partial mastectomy specimen revealed residual invasive ductal carcinoma on specimen radiograph, present as scattered tumor cell clusters measuring up to 5 mm within a tumor bed of 1.2 cm, consistent with treatment‐related change (Figure 9). Surgical resection margins were negative for invasive or in situ ductal carcinoma on final pathology report and the specimen included the former biopsy site change, the two SmartClips™ and tissue marker. Within the completion left lymphadenectomy specimen, there was one additional node containing cancer cells in addition to the initial targeted node removed using the SmartClip™ localization among the 28 nodes removed.

## 4 DISCUSSION

Use of the EnVisio™ system, with precise distances of the operating electrocautery unit to the localized SmartClips™ enabled a complex case to be approached with greater confidence. This was reinforced by the ability of the surgeon to continually monitor the distance of the operating electrocautery unit relative to the Smartclip™ of concern. Intraoperative radiographs of specimens removed that were localized would be performed in the context of any localization method, but that feedback is only upon completion of the dissection.^15^ The main challenge using this technique relates to the potential vertical alignment of the colored shapes that represent the SmartClip™ locations. Here, with the shifting of the patient's large breast to the side of her body, the two brackets anterior and posterior to the cancer were nearly over the Smartclip™ localizing the left axillary lymph node initially biopsied by core needle. Yet the obscured indicators were easily separated on the HUD by lifting the breast and pulling it more medial. This positioning was then maintained while excising the tissues localized by Smartclips™.

Traditionally, this case would have been performed as a multisite needle localization the morning of the surgery. The coupling of radiology and surgery schedules has led to significant stress for both disciplines in the recent past, given the increased push for time efficiency to reduce escalating costs. As in the case of a complicated needle localization, the radiologist may feel pressure to perform the procedure quickly to accommodate the surgical schedule. Multiple imaging modalities would have been necessary in this case to bracket the malignant calcifications under mammographic guidance and to localize the lymph node under ultrasound guidance. The complexity of placing three wires can require extra time in planning the procedure on the morning of surgery.^15^ Wires used for localization have few hash markings to indicate distance, leaving the surgeon to estimate the distances needed from mammographic films taken after localization. Surgical teams can experience downtime as they wait for a patient delayed due to complicated localizations, patient transport issues, additional registration requirements, and perioperative protocols. Operating room availability and staffing issues bring time management efficiency to the forefront, and unnecessary delays result in lost efficiency and lost revenue.

A primary advantage of wire‐free localization methods is uncoupling of the radiology and surgery schedules. Radioactive iodine‐125 seed localization (RSL), and SAVI Scout® radar (SSR) inserts demonstrate significantly less patient delay preoperatively as compared to wire localization.^16^ These localization techniques compare favorably to insertion of the SmartClip™ given that they can be performed in advance of the surgical date and utilize the similar insertion methods. Some patients prefer to avoid radioactivity, as verbalized to several of the authors, although a specific study has not documented this. Use of the RSL has a limiting half‐life and is typically placed 0–5 days prior to surgery.^17^ As there is not separate designation for diagnostic use, the radioactive iodine‐125 seed is regulated in the United States by the Nuclear Regulatory Commission for therapeutic use. Strict guidelines must be followed, including an inventory of all sources. Seed loss, mishandling, or damage must be reported and can result in the institution's loss of license for radioactivity.^18^ This risk does not apply to a Smartclip™, which has no radioactivity or power source within it. It has been approved by the FDA for indefinite indwelling after placement.^19^

SAVI Scout® radar inserts in a similar fashion to the other seed type methods and the wire localization method.^9^ Relative to wire localization and radioactive seeds, this method results in a positive margin that is comparable to that of wire localization.^9^, ^20^ The radioactive seeds also appear to be comparable to wire localization in multiple hands^16^, ^17^ as well; however, the margin status (positivity) appears to vary significantly from institution to institution, ranging 5%–20%. Although published data is lacking on how the SmartClip™ compares relative to margin status, a preliminary review of our collective data (University of Cincinnati, three surgeons), suggests that it too is comparable.

In addition, radiofrequency identification tags (RFID) (LOCalizer™ radiofrequency identification, Hologic, Inc.; Marlborough, MA), Magseed® (Endomagnetics, Inc., Austin, TX), and SmartClip™ are all seed‐type wireless localizers placed independently of the day of surgery, resulting in fewer delays on the day of surgery. Patient comfort is improved on the day of localization without the added stress of surgery and NPO status. The patient is also more comfortable the day of surgery since there are no external components for the patient to manage as with wire localizations. Placement of a wire in the axilla can be particularly uncomfortable for patients and subject to migration in the duration between placement and excision due to fidgeting. These seed type wireless localizers all avoid the risk of complications associated with an external wire component and do not impact the surgical approach to excision or the location of the incision.

Studies published that examine resection margin status in using wire‐guided localizations, RSL and SSR, Magseed® and RFID appear to be comparable or slightly improved over wire localization in obtaining negative margins on excision.^20^, ^21^, ^22^, ^23^, ^24^, ^25^, ^26^, ^27^, ^28^ The technical difficulties in placing these seeds are similar to tissue marker placement, with the risk of seed migration, failure of seed placement, and inadvertent deployment of seed.^29^, ^30^, ^31^ Given similar methods of deployment, it is not surprising that the similar technical difficulties result.

This article describes localization and successful excision with more than one SmartClip™ seed. With some implanted localization devices, differentiating between the seed locations could become a challenge. This article describes the use of three separate SmartClips™ placed and excised without difficulty; one of these clips was used to tag a lymph node previously marked and known to be positive for metastatic disease prior to neoadjuvant chemotherapy. Certainly, multiple localizations have been described using wire localizations,^32^ as well as radioactive seeds.^33^, ^34^ Others have described the use of radar reflectors^35^ or magnetic seeds,^36^ and other groups have described the use of tagging a previously marked positive lymph node following neoadjuvant chemotherapy.^37^ Yet very few reports on wireless localizers describe the use of two or more seeds in this context.^26^, ^38^

In the shift toward increasing use of neoadjuvant therapy prior to definitive surgical management of breast cancer, wireless localizations are playing an increasingly important role in the localization of biopsy‐proven metastatic axillary lymph nodes in patients with breast cancer following systemic therapy. In light of our experience, up to three SmartClips™ could be inserted at the time of biopsy, or closer to surgical date. To our knowledge, this is the first report of use of the SmartClip™ for localization, and only one of a handful of reports utilizing three sites of localization. Yet, three localizations are no longer rare in this context and use of the SmartClip™ and its clear differentiation from other sites made the case straightforward.

SmartClips™ are approved by the FDA for both temporary and permanent insertion within the breast tissue.^19^ Theoretically, such a seed could be placed at the time of biopsy to facilitate eventual surgical resection in cases of high suspicion for breast cancer. However, MRI assessment of the presenting breast cancer and possibly its response to neoadjuvant therapy may be desired in this context. Both the SmartClip™ and RFID cause up to 2 cm of susceptibility artifact, with larger artifact using the Magseed®.^18^ This artifact can significantly limit evaluation of residual disease and response to treatment.

## 5 CONCLUSION

The use of the SmartClip™ fiducial proved successful in its application to the setting of multiple localizations for surgical excision. The continuous feedback to the surgeon as to resection location relative to the Smartclip™ position easily enabled a timely excision of multiple sites.

## AUTHOR CONTRIBUTIONS

**Elizabeth A. Shaughnessy:** Conceptualization (equal); data curation (equal); formal analysis (equal); investigation (equal); methodology (equal); visualization (equal); writing – original draft (equal); writing – review and editing (equal). **Charmi Vijapura:** Investigation (equal); methodology (equal); writing – original draft (equal); writing – review and editing (equal). **Chantal Reyna:** Conceptualization (equal); formal analysis (equal); investigation (equal); methodology (equal); writing – original draft (equal); writing – review and editing (equal). **Jaime Lewis:** Conceptualization (equal); methodology (equal); writing – original draft (equal); writing – review and editing (equal). **Kyle Lewis:** Methodology (equal); writing – original draft (equal); writing – review and editing (equal). **Su‐Ju Lee:** Methodology (equal); writing – original draft (equal). **Lawrence Sobel:** Methodology (equal); writing – original draft (equal); writing – review and editing (equal). **Rifat Wahab:** Methodology (equal); writing – original draft (equal); writing – review and editing (equal). **Lauren Rosen:** Visualization (equal); writing – original draft (equal); writing – review and editing (equal). **Ann Brown:** Conceptualization (equal); data curation (equal); formal analysis (equal); investigation (equal); methodology (equal); visualization (equal); writing – original draft (equal); writing – review and editing (equal).

## CONFLICT OF INTEREST

Dr. Chantal Reyna recently became a consultant for Elucent Medical. The remaining authors have explicitly stated that there are no conflicts of interest in connection with this article.

## ETHICS STATEMENT

The study was approved by the Institutional Review Board of the University of Cincinnati (protocol #2020‐0675) and written informed consent was obtained.

## Data Availability

Data sharing not applicable ‐ no new data generated.
